# Research on Glass Frit Deposition Based on the Electrospray Process

**DOI:** 10.3390/ma9040292

**Published:** 2016-04-18

**Authors:** Yifang Liu, Daner Chen, Zhan Zhan, Chenlei Li, Jianyi Zheng, Daoheng Sun

**Affiliations:** School of Aerospace Engineering, Xiamen University, Xiamen 361005, China; yfliu@xmu.edu.cn (Y.L.); chendaner@stu.xmu.edu.cn (D.C.); zhanzhan_snow@163.com (Z.Z.); lichenlei@stu.xmu.edu.cn (C.L.); zjy@xmu.edu.cn (J.Z.)

**Keywords:** glass frit, film, patterned deposition, electrospray

## Abstract

In this paper, the electrospray technology is used to easily deposit the glass frit into patterns at a micro-scale level. First, far-field electrospray process was carried out with a mixture of glass frit in the presence of ethanol. A uniform, smooth, and dense glass frit film was obtained, verifying that the electrospray technology was feasible. Then, the distance between the nozzle and the substrate was reduced to 2 mm to carry out near-field electrospray. The experimental process was improved by setting the range of the feed rate of the substrate to match both the concentration and the flow rate of the solution. Spray diameter could be less at the voltage of 2 kV, in which the glass frit film was expected to reach the minimum line width. A uniform glass frit film with a line width within the range of 400–500 μm was prepared when the speed of the substrate was 25 mm/s. It indicates that electrospray is an efficient technique for the patterned deposition of glass frit in wafer-level hermetic encapsulation.

## 1. Introduction

Glass frit bonding is a widely used wafer-level hermetic encapsulation technology due to its wide choice of cover materials, low sealing temperature, high tolerance for roughness of bonding interface, and possible electrical feedthroughs on the bonding surface [1,2,3]. Patterned glass frit is conventionally deposited via screen printing [1]. Although the advantages of the screen printing method include process simplicity, low production cost, and variable preparation area [4], the pattern-distortion-vulnerability and material-wasting make it incapable of meeting the requirement of the package of MEMS sensors.

Electrospray is a typical process of jet spray [5] using liquids of low viscosity, which has been investigated since the 1960s for applications in paint, insecticides, inks, and xerographic liquid [6]. In a typical electrospray system, a liquid is fed through a capillary and is charged at a high voltage relative to a nearby ground electrode. Within the interaction of Coulomb repulsion and surface tension, the liquid would deform locally to an elongated jet and subsequently break into dispersed droplets with a tunable diameter ranging from a few nanometers to hundreds of micrometers [7,8]. Spraying parameters including the flow rate of liquid, the electrical potential, and the diameter of the nozzle should be taken into consideration, as well as the material properties [9,10,11,12,13,14,15]. The advantages of the electrospray technique include simple operation, an efficient process, an evenly deposited layer, and good compatibility of materials [9,16]. The electrospray has been developed to prepare a diverse range of thin films of biomaterial, composite metal, and nonmetal oxides, such as SiO_2_ [17,18,19], TiO_2_ [20], ZnO [21,22], and SnO [23]. The main ingredient of glass frit is SiO_2_, and electrospray is expected to replace screen printing to obtain precise positioning of the glass frit layer with uniform width. Kazuhiko Higashi *et al.* have demonstrated that the micropatterning of silica nanoparticles can be realized by electrospray deposition [24]. However, there are few reports on the research of the electrospraying of glass frit. Herein, the patterned deposition of glass frit using the electrospray process is proposed in this paper, and the achievement of micro-scale glass frit patterns is investigated.

## 2. Materials and Methods

### 2.1. Experimental Facilities

**Figure 1:**
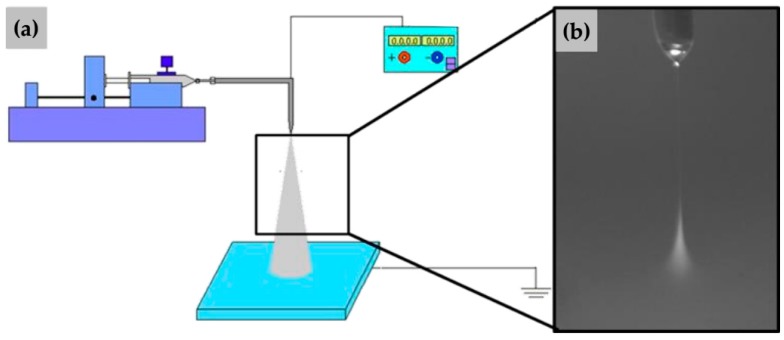
Schematic of the electrospray setup: (**a**) fundamental setup; (**b**) spray plume.

The experimental setup is schematically shown in Figure 1, mainly consisting of a stainless-steel capillary nozzle (inner diameter = 0.34 mm), a high-voltage power supply (DW-P503-1ACDE), a precise syringe pump (Pump 11 Elite, Harvard Apparatus, Holliston, MA, USA), and a silicon collector (diameter = 500 mm and thickness = 0.5 mm). The nozzle was mounted on a syringe propelled by the precise syringe pump. Meanwhile, this nozzle was connected to the positive electrode of a high-voltage power supply, and the silicon collector was grounded. The electrospray process was recorded by using a CCD camera (UI-2250SE-C-HQ, IDS, Obersulm, Germany). The deposited pattern was examined with a scanning electron microscope (SU-70, HITACHI, Tokyo, Japan) and an optical microscope (Custom made, Mitutoyo, Tokyo, Japan).

### 2.2. Materials

The solid content of the glass frit used in our experiments was 80% (purchased from Xiamen University-Parkathings Integrated Application Research Center of Rare Earth, Product No. VH 853, Xiamen, China), among which 90% of its particles were smaller than 16.35 μm, and the average particle size was 5.70 μm. To prepare the homogeneous solution for electrospray, ethanol was added to the glass frit. Then, the suspension was stirred by a magnetic stirrer (C-MAG HS7, IKA, Staufen, Germany) for 3 h at room temperature until a uniform solution was obtained. As white precipitate would appear after standing for 15 min, the solution was stirred for 30 min before each use, in order to maintain relative stability over the duration of the deposition. The substrate silicon wafer (4 inches) was commercially available, with one side polished, <100> oriented, N-type and 1–10 Ω cm resistivity.

### 2.3. Details of Far-Field Electrospray Experiments

In previous researches, Jungmyoung Ju *et al.* and Takeshi Fukuda *et al.* demonstrated that the relative dielectric constant of the organic solvent plays an important role on the spray diameter, and the vapor pressure of the second organic solvent is a dominant factor for smooth surface [25,26]. On the basis of material properties, ethanol was chosen as the solvent. Solutions with different glass frit *versus* ethanol mass ratios were used in the far-field electrospray experiments. In the orthogonal test, the ranges of the concentration of the glass frit solution, the applied voltage, and the flow rate of solution were 10%–20%, 7.5–9.5 kV, 750–1250 μL/h, respectively, as shown in Table 1. The distance between the nozzle and the silicon substrate was fixed at 11.5 cm. The electrospray period for each sample was 120 s.

### 2.4. Details of Near-Field Electrospray Experiments

In this section, the flow rate of the solution was set to 100 μL/h. The distance between the nozzle and the substrate was 2 mm. The applied voltage was regulated from 0 to 4 kV. The concentration of the glass frit solution was 10 wt % with ethanol being the solvent.

In order to attain the linear glass frit film with the width of microscale, the substrate was moved at different speeds of 6, 10, 15, 20, and 25 mm/s. The applied voltage, the concentration of the glass frit solution, the flow rate of the solution, and the distance between the nozzle and the substrate were fixed at 2 kV, 10 wt %, 100 μL/h, and 2 mm, respectively. Each film sample was sprayed for 5 min.

## 3. Results and Discussion

### 3.1. The Stability of Spray Plume and the Deposition Profile of Aerosol

Previous literature [27,28] divides the electrospray process into a variety of classifications such as dripping mode, micro-dripping mode, spindle mode, cone-jet mode, oscillation mode, and multi-jet mode. Among these modes, the cone-jet mode is stable and has the feasibility of obtaining uniform droplets. In order to verify the feasibility of electrospray in defining glass frit patterns, the orthogonal test under the traditional far-field electrospray method was conducted first. Experimental parameters including the concentration of glass frit solution, the applied voltage, and the flow rate of solution were taken into consideration.

**Figure 2:**
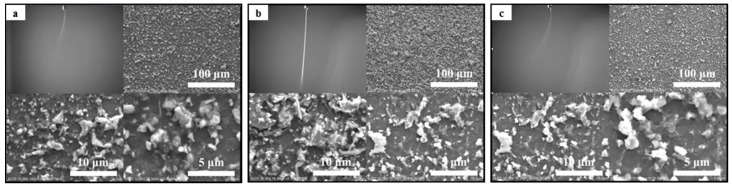

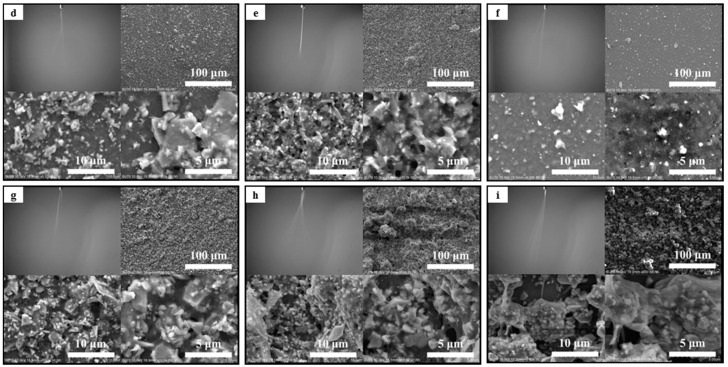
Images of spray plume and deposition film on silicon substrate (glass frit in ethanol, inner diameter of capillary nozzle 340 μm, electrode gap 11.5 cm, deposition time 120 s): (**a**) concentration 10 wt %, voltage 7.5 kV, flow rate 750 μL/h; (**b**) concentration 10 wt %, voltage 8.5 kV, flow rate 1000 μL/h; (**c**) concentration 10 wt %, voltage 9.5 kV, flow rate 1250 μL/h; (**d**) concentration 15 wt %, voltage 7.5 kV, flow rate 1000 μL/h; (**e**) concentration 15 wt %, voltage 8.5 kV, flow rate 1250 μL/h; (**f**) concentration 15 wt %, voltage 9.5 kV, flow rate 750 μL/h; (**g**) concentration 20 wt %, voltage 7.5 kV, flow rate 1250 μL/h; (**h**) concentration 20 wt %, voltage 8.5 kV, flow rate 750 μL/h; (**i**) concentration 20 wt %, voltage 9.5 kV, flow rate 1000 μL/h.

Figure 2 shows the images of jets in the electrospray process as well as the corresponding SEM images of nine deposited patterns (Figure 2a–i correspond to the experimental parameters of Tests 1–9 in Table 1). From the result of Test 1 shown in Figure 2a, we can see that a stable jet formed under the experimental conditions of Test 1 and that the deposited particles distributed evenly, but the amount of particles was too small. This is because, when all three experimental parameters including the viscosity of the solution, the flow rate of the solution, and the applied voltage are set low, the single jet would scatter into many branches to make the spray angle too large.

In Figure 2b–d, the spray plumes are unstable, some even deflect. The deposited layers contain clusters and dispersed particles of different diameters, which could be explained by the fact that both the applied voltage and the flow rate of the solution did not match the concentration of the solution. At low concentrations of the solution, both the relative high voltage and flow rate cause the droplets to distribute in multi-polarization in the atomization zone. In this case, the amount of charge carried by the droplets was uneven, resulting in Coulomb repulsion between the particles of different droplets, eventually leading to the varied particles and incompact glass frit layers.

Spray jets in Figure 2f branch off within short distances away from the outlet of the nozzle such that the deposited particles spread out and are not closely packed. In this case, the length of the jet region decreased when the flow rate of the solution was slow. On the other hand, the amount of charge was large when the applied voltage was high. Therefore, the jet quickly broke into multi-jets leading to the large and unstable atomizing zone to form the poor deposition of glass frit.

The result of Test 7 is indicated in Figure 2g. Under these conditions, the bifurcation of jet occurred and large droplets dripped intermittently to make the deposited layer uneven. This can be explained by the fact that the charge accumulates in a drop-like jet and the jet finally breaks into droplets under both the high concentration of glass frit solution (20 wt %) and the flow rate of the solution.

Figure 2h shows the uneven layers consisting of agglomerates. The jet area decreased due to the low flow rate, and the relatively high voltage made the droplets atomize rapidly. The atomization process became very messy; some of the droplets scattered, while others floated in air. The jet was very unstable and a poor atomization was attained.

The fact that the atomization region was not stable under the experimental conditions of Test 9 can be inferred from Figure 2i. During the spraying process, large droplets are formed and fall intermittently, and the sizes of deposited particles differ greatly. Thus, the atomized particles cannot be tightly packed to form a continuous and dense glass frit layer.

The flat glass frit layer in Figure 2e is dense with uniform particles distributed compactly. Under these conditions, a stable cone flow is formed. This is due to the fact that, under the action of both the applied voltage and the flow rate, the liquid of the specific viscosity does not break early. At a certain distance from the nozzle, the single jet becomes a scattering-like multi-jet, and the cone atomization is formed in the diffusion region. A high flow rate ensures that the atomizing angle is not too large to form a uniform and dense distribution of the particles.

The results demonstrate that the concentration of the solution mainly affects the density of the deposition layer, while the applied voltage has an impact on the stability and break of the jet, and the flow rate of the solution determines the region of atomization. Additionally, the deposited glass frit layer can be more uniform, smooth and dense, with an electrode gap of 11.5 mm, a solution density of 15%, a voltage of 8.5 kV, and a flow rate of 1250 μL/h.

### 3.2. Preparation of Micro-Scale Linear Film

For traditional far-field electrospray, the deposition area as well as the width of deposited patterns usually ranges from several centimeters to dozens of centimeters due to the large gap between the nozzle and the silicon substrate. It should be desired to minimize the deposition area to meet the requirement of the development of spraying technology, especially the emerging micro/nano manufacture. To obtain micro-scale patterns, a near-field electrospray technique was investigated by reducing the distance between the nozzle and the substrate to several millimeters.

**Figure 3:**
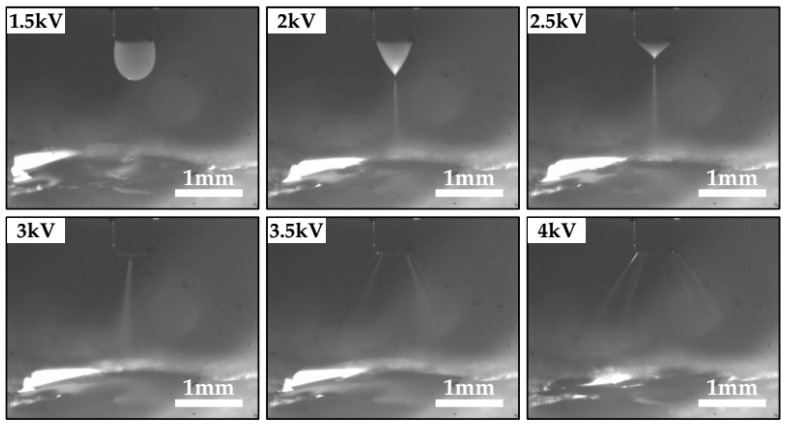
Photographs of the spray jet in near-field electrospray with varying applied voltages (solution concentration 10 wt %, flow rate 100 μL/h, electrode gap 2 mm).

In electrospraying, the flowing liquid is forced by the electric field to be dispersed into fine droplets [10]. The electric field is related to the applied voltage and the distance between nozzle and ground substrate. When the distance becomes 2 mm, the voltage should be regulated to match up with the distance to generate a stable cone-jet mode. Figure 3 shows the CCD images of electrospray jet in the near-field experiments with varying applied voltages.

When the applied voltage is in the range of 0–1.8 kV, the electrical force subjected to the liquid is insufficient to induce the electrospray process, such that the liquid droplet would fall under the force of gravity. By increasing the applied voltage, stable cone-jet mode can be obtained in the range of 1.8–2.6 kV. When the voltage reaches 3 kV, a stable cone-jet mode shifts into oscillation mode. The spray jet is no longer straight and inclines towards a random direction. This is closely related to the severe oscillation of the jet before it reaches the silicon substrate. The multi-jet mode appears at an applied voltage higher than about 3.4 kV. Furthermore, a corona discharge can be seen between the nozzle and the substrate when the applied voltage exceeds 4 kV. As a result, the electrospray process is no longer maintained. In stable cone-jet mode, the solution is dispersed into fine droplets a little earlier at 2.5 kV than at 2 kV. Spray diameter can be less at the condition of 2 kV. Therefore, the glass frit film is expected to reach the minimum line width at the applied voltage of 2 kV.

**Figure 4:**
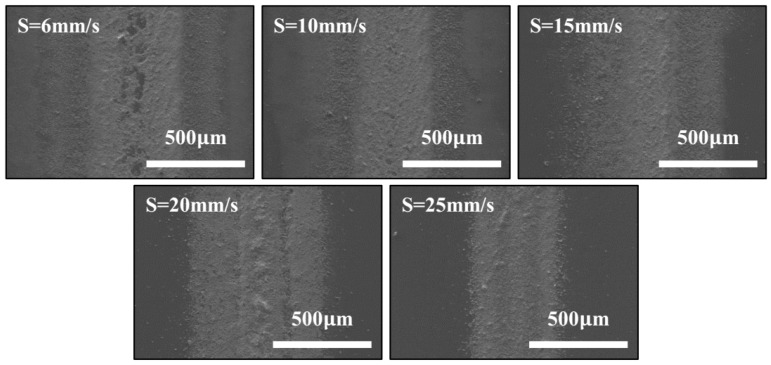
Photographs of linear glass frit film at different motion speeds (*S*) of the substrate (6, 10, 15, 20, and 25 mm/s).

On the basis of the above researches, the applied voltage, the concentration of the glass frit solution, the flow rate of the solution and the distance between the nozzle and the substrate were fixed at 2 kV, 10 wt %, 100 μL/h, and 2 mm, respectively, so as to produce a linear glass frit film with the width of a microscale. The substrate was moved at different speeds of 6, 10, 15, 20, and 25 mm/s. Each film sample was sprayed for 5 min. Figure 4 reveals that the smaller width of the glass frit pattern can be obtained at the motion speed of the substrate of 25 mm/s, and the width of the deposited film is 438 μm. Additionally, the patterned film is denser than others. At the speed of 6 mm/s and 10 mm/s, the residual solvent of the deposited patterns flows on the silicon substrate, which makes up approximately half of the linear film. When the speed rises up to 15 mm/s and 20 mm/s, the area of the residual solvent narrows, and the width of the patterned glass frit reduces to about 700 μm. This may be explained by the fact that the spray jet is stretched towards the motion direction at a faster motion speed of the substrate, and the depositing particles tend to cluster together to the center of the glass frit film.

**Figure 5:**
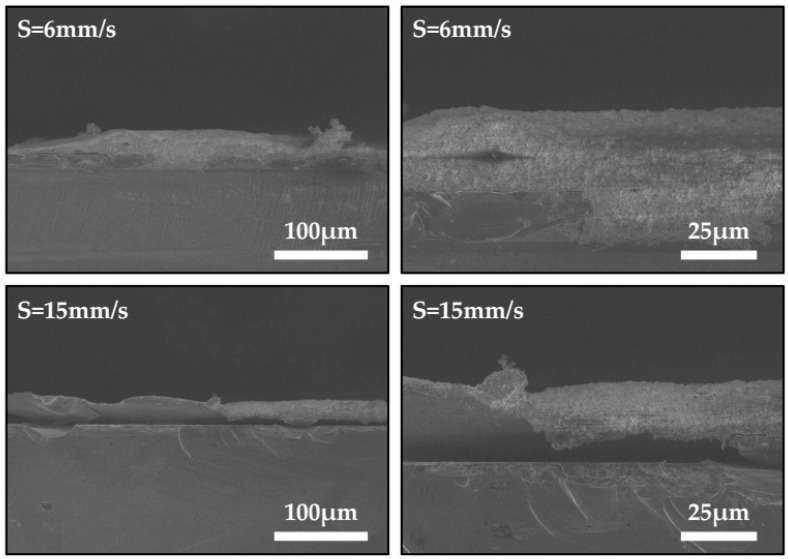
Images of the thickness profiles across the linear films at speeds (*S*) of 6 mm/s and 15 mm/s.

**Figure 6:**
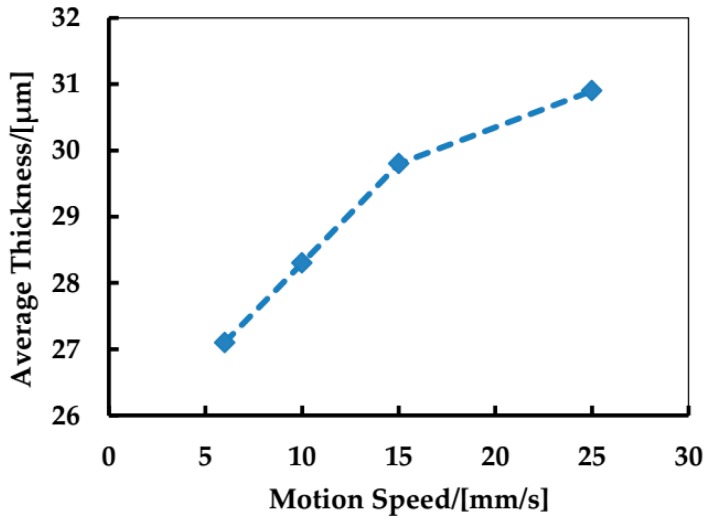
The average thickness profiles of the glass frit films at different speed of the substrate (6, 10, 15, 25 mm/s).

For further demonstration, the thickness profiles across the linear films were measured using a scanning electron microscope. Figure 5 shows the results of the thickness profiles at speeds of 6 mm/s and 15 mm/s. The samples here were sprayed for 20 min, for the sake of distinct thickness. The average thickness of the glass frit films at different speeds was measured, as shown in Figure 6. As the motion speed of the substrate changes from 5 to 25 mm/s, the average thickness increases from 27 to 31 μm. Consequently, the glass frit film appears narrower at a higher motion speed because these films are generally thicker due to less material flowing towards both sides. The thickness profiles of the glass frit films could be useful for the patterned deposition of glass frit in wafer-level hermetic encapsulation.

## 4. Conclusions

Electrospray of glass frit solution is investigated in this paper. The results show that relatively uniform, smooth, and dense glass frit film can be obtained from the far-field electrospray process, with an electrode gap of 11.5 mm, a solution density of 15%, a voltage of 8.5 kV and a flow rate of 1250 μL/h. Thus, it has here been proven that electrospraying is suitable for patterned glass frit deposition. Furthermore, the spray mode varies with the increasing applied voltage in the near-field electrospray process. Stable cone-jet mode and a shorter spray diameter appear at the applied voltage of 2 kV, and the glass frit film is expected to reach a minimum line width. The width of the deposited glass frit film changes with the feed rate of the substrate. Linear film with line width within the range of 400–500 μm is prepared under the experimental conditions where the electrode gap is 2 mm, the voltage is 2 kV, the flow rate is 100 μL/h, the motion speed is 25 mm/s, and the solution density is 10%. It can be concluded that electrospraying is a novel technique for the patterned deposition of glass frit. Further studies will emphasize the thickness of patterned glass frit and its practical applications.

## Figures and Tables

**Table 1:** Table of L9(3^3^) orthogonal test.

Test No.	Glass Frit Concentration/(wt %)	Voltage/(kV)	Flow Rate/(μL/h)	Result
1	10	7.5	750	Figure 2a
2	10	8.5	1000	Figure 2b
3	10	9.5	1250	Figure 2c
4	15	7.5	1000	Figure 2d
5	15	8.5	1250	Figure 2e
6	15	9.5	750	Figure 2f
7	20	7.5	1250	Figure 2g
8	20	8.5	750	Figure 2h
9	20	9.5	1000	Figure 2i

## Abbreviations

The following abbreviations are used in this manuscript:

MEMS: Micro-electromechanical Systems
CCD: Charge Coupled Device
SEM: Scanning Electron Microscope
